# Immune Response of Eastern Honeybee Worker to *Nosema ceranae* Infection Revealed by Transcriptomic Investigation

**DOI:** 10.3390/insects12080728

**Published:** 2021-08-14

**Authors:** Wenhao Xing, Dingding Zhou, Qi Long, Minghui Sun, Rui Guo, Limei Wang

**Affiliations:** 1 College of Animal Science, Guizhou University, Guiyang 550025, China; xingwenhao2021@126.com; 2 College of Animal Sciences (College of Bee Science), Fujian Agriculture and Forestry University, Fuzhou 350002, China; zdd03569981@163.com (D.Z.); l1070190695@126.com (Q.L.); hnmhsun@126.com (M.S.); 3 Apitherapy Research Institute, Fujian Agriculture and Forestry University, Fuzhou 350002, China; 4 Dongying Vocational Institute, Dongying 257000, China; wlm11@163.com

**Keywords:** honeybee, *Apis cerana*, *Apis cerana cerana*, *Nosema ceranae*, immune response

## Abstract

**Simple Summary:**

Currently, knowledge regarding *Apis cerana*–*Nosema ceranae* interaction is very limited, though *A. cerana* is the original host of *N. ceranae*. *Apis cerana cerana* is a subspecies of *A. cerana* and a major bee species used in the beekeeping industry in China and other countries. Here, the effective infection of *A. c. cerana* workers by *N. ceranae* was verified, followed by transcriptomic investigation of host responses. Furthermore, immune responses between *A. c. cerana* and *Apis mellifera ligustica* were deeply compared and discussed. In total, 1127 and 957 *N. ceranae*-responsive genes were identified in the infected midguts at 7 d post-inoculation (dpi) and 10 dpi, respectively. Additionally, DEGs in workers’ midguts at both 7 dpi and 10 dpi were associated with six cellular immune pathways and three humoral immune pathways. Noticeably, one up-regulated gene was enriched in the NF-*κ*B signaling pathway in the midgut at 10 dpi. Further analysis indicated that different cellular and humoral immune responses were employed by *A. c. cerana* and *A. m. ligustica* workers to combat *N. ceranae*. Our findings provide a foundation for clarifying the mechanisms regulating the immune response of *A. c. cerana* workers to *N. ceranae* invasion and developing new approaches to control bee microsporidiosis.

**Abstract:**

Here, a comparative transcriptome investigation was conducted based on high-quality deep sequencing data from the midguts of *Apis cerana cerana* workers at 7 d post-inoculation (dpi) and 10 dpi with *Nosema ceranae* and corresponding un-inoculated midguts. PCR identification and microscopic observation of paraffin sections confirmed the effective infection of *A. c. cerana* worker by *N. ceranae*. In total, 1127 and 957 *N. ceranae*-responsive genes were identified in the infected midguts at 7 dpi and 10 dpi, respectively. RT-qPCR results validated the reliability of our transcriptome data. GO categorization indicated the differentially expressed genes (DEGs) were respectively engaged in 34 and 33 functional terms associated with biological processes, cellular components, and molecular functions. Additionally, KEGG pathway enrichment analysis showed that DEGs at 7 dpi and 10 dpi could be enriched in 231 and 226 pathways, respectively. Moreover, DEGs in workers’ midguts at both 7 dpi and 10 dpi were involved in six cellular immune pathways such as autophagy and phagosome and three humoral immune pathways such as the Toll/Imd signaling pathway and Jak-STAT signaling pathway. In addition, one up-regulated gene (XM_017055397.1) was enriched in the NF-*κ*B signaling pathway in the workers’ midgut at 10 dpi. Further investigation suggested the majority of these DEGs were engaged in only one immune pathway, while a small number of DEGs were simultaneously involved in two immune pathways. These results together demonstrated that the overall gene expression profile in host midgut was altered by *N. ceranae* infection and some of the host immune pathways were induced to activation during fungal infection, whereas some others were suppressed via host–pathogen interaction. Our findings offer a basis for clarification of the mechanism underlying the immune response of *A. c. cerana* workers to *N. ceranae* infection, but also provide novel insights into eastern honeybee-microsporodian interaction.

## 1. Introduction

Honeybees are a pivotal pollinator for a great number of wild plants and crops throughout the world [1]. In addition, honeybees are widely used as a model for investigating caste differentiation [2], social behavior [3], disease transmission [4], epigenomics [5], gene regulation [6], and recently, transgenic manipulation [7] and gene editing [8].

However, as a eusocial insect, honeybees are vulnerable to infection by many pathogens and parasites. Among them, *Nosema ceranae* is a spore-forming unicellular and obligate intracellular fungal parasite that exclusively infects the midgut epithelium cells and not all epithelial cells of the adult honeybee, leading to bee microsporidiosis [9,10]. *N. ceranae* was first identified in 1996 in colonies of the Asian honeybee, *Apis cerana* [11]. Evidently, it switched hosts from *A. cerana* to *Apis mellifera* some decades ago and spread to western honeybee colonies throughout the world [12]. Currently, *N. ceranae* is the predominant microsporidian infecting bees with a worldwide distribution [13,14]. *N. ceranae* infection has been suggested to affect the health of honeybee hosts via disturbance of hormones [15], reduction of lifespan [16], degeneration of midgut epithelial cells [17], energetic stress [18], cell apoptosis inhibition [19,20,21], and immunity suppression [22,23].

Honeybees have developed various defense mechanisms against parasitic infection, including cellular and humoral immune responses [24]. The honeybee humoral response has been shown to involve four antimicrobial peptides: abaecin, apidaecin, defensin, and hymenoptaecin [25]. In the past decade, the impact of *N. ceranae* on immune defense of the western honeybee has been widely studied [15,23,26,27,28,29]. Our group previously performed next-generation sequencing and transcriptome analysis of *Apis mellifera ligustica* workers’ midguts infected by *N. ceranae*, and found that both cellular and humoral immune responses were activated during the early infection stage, that the former may play a major role in defense against fungal infection, and that host cellular immune response was increasingly enhanced during the late stage of infection while the humoral immune response weakened significantly [30]. Meanwhile, we revealed the mechanism underlying *N. ceranae* infection of *A. m. ligustica* workers by analyzing the differential expression profile of fungal genes [31].

*A. cerana* is endemic to Asia and has been used for commercial beekeeping and pollination over thousands of years [32]. *A. cerana* differs from *A. mellifera* in distinct biological characteristics, including longer flight duration [33], effective grooming and hygienic behaviors [34], adaptation to extreme weather conditions [35], lower production rate of royal jelly [36], and cooperative group-level defenses [37]. The reference genome of *A. cerana* was published in 2015; it allows deep investigation of the eastern honeybee’s biology including immunity and development. Currently, study on *A. cerana*-*N. ceranae* interaction has lagged significantly, although *A. cerana* is the primary host of *N. ceranae*. Compared to the western honeybee, knowledge of the eastern honeybee’s immune response to *N. ceranae* invasion is very limited.

To analyze the cellular and humoral immune responses of *A. c. cerana* workers to *N. ceranae* infection, comparative transcriptome investigation was conducted in this work based on previously obtained high-quality deep sequencing data from midguts of *A. c. cerana* workers at 7 d post inoculation (dpi) and 10 dpi with *N. ceranae* and corresponding un-inoculated midguts. Findings from our study not only offer a foundation for clarification of the molecular mechanism regulating the immune response of *A. c. cerana* workers to *N. ceranae* infection, but also provide novel insights into eastern honeybee-microsporidian interaction during bee nosemosis.

## 2. Materials and Methods

### 2.1. Transcriptome Data Source

Three *N. ceranae*-free colonies of *A. c. cerana* located in the teaching apiary of the College of Animal Sciences (College of Bee Science) in Fujian Agriculture and Forestry University were selected for the present study. Before and during the whole experiment, no *Varroa* was detected. RT-PCR was performed to examine the prevalence of two bee microsporidia (*N. ceranae* and *Nosema apis*) and seven common bee viruses (ABPV, BQCV, CBPV, DWV, KBV, IAPV, and SBV) in the newly emergent *A. c. cerana* workers using previously developed specific primers (Table S1) [38,39,40,41,42,43,44]. Agarose gel electrophoresis (AGE) showed that no signal bands were amplified from the above-mentioned two microsporidia and seven viruses (Figure S1).

*A. c. cerana* workers’ midguts inoculated with *N. ceranae* and corresponding un-inoculated midguts were prepared as described previously [45]. Briefly, (1) the midguts of 50 infected workers from one colony located in Yuan’an apiary in Fuzhou city were removed and crushed in sterile water and filtered with four layers of gauze, centrifuged at 6000× *g* for 5 min, and then purified on a discontinuous Percoll gradient; a small quantity of spores were identified and verified to be mono-specific using PCR with previously developed primers (F: CGGATAAAAGAGTCCGTTACC; R: TGAGCAGGGTTCTAGGGAT) [39], followed by measurement of spore concentration with a CL kurt counter (JIMBIO). (2) Newly emerged *Nosema*-free workers were carefully removed from frames from three *A. c. cerana* colonies, kept in plastic cages in groups of 30, and reared in an incubator at 34 ± 2 °C for 24 h after eclosion. The workers in *N. ceranae*-treated groups (*n* = 3) were starved for 2 h, and then each was fed 5 μL of a 50% sucrose (*w/v* in sterile water) solution containing 1 × 10^6^ *N. ceranae* spores [46], whereas workers in control groups (*n* = 3) were each fed 5 μL of a 50% sucrose solution without fungal spores; workers that did not consume the entire droplet were discarded. The workers were fed *ad libitum* with a solution of sucrose (50% *w/w*) for 24 h after inoculation, and the feeders were replaced daily throughout the whole experiment. Each cage was checked every 24 h, and dead bees were removed. (3) Nine workers from each cage in the *N. ceranae*-treated and control groups were sacrificed at 7 d post-inoculation (dpi) and 10 dpi, followed by dissection and separation of the midgut samples, which were immediately pooled and frozen in liquid nitrogen.

Total RNA extraction, cDNA library construction, and strand-specific cDNA library-based RNA-seq of the midgut samples were conducted as previously described [47]. In brief, total RNA of the aforementioned 12 midgut samples was respectively isolated using Trizol (Life Technologies) and detected using 1% AGE. After removal of rRNAs, mRNAs were fragmented into short fragments using fragmentation buffer (Illumina) and then reverse-transcribed into cDNA with random primers. Second-strand cDNA was synthesized and then purified, end repaired, poly (A) added, and ligated to Illumina sequencing adapters, followed by digestion of the second-strand cDNA. The digested products were size selected by AGE, PCR amplified, and sequenced on illumina HiSeq^TM^ 4000 system (illumine, San Diego, CA, USA) by Guangzhou Gene Denovo Biotechnology Co. We used AcCK1-1, AcCK1-2, and AcCK1-3 as three replicate libraries for midgut samples at 7 dpi in control groups, AcT1-1, AcT1-2, and AcT1-3 as three replicate libraries for midgut samples at 7 dpi in *N. ceranae*-treated groups, AcCK2-1, AcCK2-2, and AcCK2-3 as three replicate libraries for midgut samples at 10 dpi in control groups, and AcT2-1, AcT2-2, and AcT2-3 as three replicate libraries for midgut samples at 10 dpi in *N. ceranae*-treated groups.

Quality control of the generated raw reads and mapping of the clean reads were previously performed [45] according to the method described by Chen et al. [47]. In short, raw reads were filtered to gain clean reads with high quality by removing reads containing adapters, more than 10% of unknown nucleotides (N), and more than 50% of low quality (*q* value ≤ 20) bases, which were then aligned to the ribosome RNA (rRNA) database (www.arb-silva.de/) (20 March 2021) with Bowtie2 [48]. Next, the rRNA-removed clean reads of each group were aligned to the *A. cerana* reference genome (assembly ACSNU-2.0) utilizing TopHat2 [49], and the mapped clean reads were then used for downstream analyses. The result of quality control demonstrated that 124,216,829, 174,700,032, 99,030,788, and 205,297,946 raw reads were produced from AcCK1, AcT1, AcCK2, and AcT2 groups, and 121,949,977, 171,868,061, 97,432,267, and 200,570,776 clean reads were obtained after quality control, with Q20 of 94.60%, 94.96%, 94.91%, and 94.58%, respectively. Additionally, 75,553,033, 68,416,248, 64,919,402, and 65,107,760 clean reads could be mapped to the reference genome of *A. cerana* (assembly ACSNU-2.0); moreover, Pearson correlation coefficient for each group was above 0.87 [44]. Taken together, the high-quality transcriptome data could be used for related analyses in this current work.

### 2.2. Confirmation of Effective Infection of Eastern Honeybee Workers by N. ceranae

Following the above-mentioned procedure, *A. c. cerana* 1-day-old workers in *N. ceranae*-treated groups were each artificially inoculated with 1 × 10^6^ *N. ceranae* spores. The midguts at 12 dpi in the *N. ceranae*-treated group and un-treated group were respectively dissected out and fixed using 4% paraformaldehyde. Paraffin sections were prepared using a microtome (Leica, Nussloch, Germany) and an embedding center (Junjie, Wuhan, China) and then stained with hematoxylin eosin (HE) stain by Shanghai Sangon Biological Engineering Co. Ltd., followed by observation using an optical microscope with digital camera (SOPTOP, Shanghai, China).

Total DNA of workers’ midgut at 12 dpi with *N. ceranae* was isolated as PCR template. Clean spores of *N. ceranae* were set as a positive control. Workers’ midgut at 12 dpi without *N. ceranae* was set as a negative control, and sterile water as another negative control. PCR amplification was conducted on a T100 thermo cycler (BIO-RAD) with previously described primers for *N. ceranae* (F: CGAGCGGTTTCCCATCTCAGTA; R: AAAACACCGGAACTCGTCAGCT) [38] and the following conditions: pre-denaturation step at 94 °C for 5 min; 37 amplification cycles of denaturation at 94 °C for 50 s, annealing at 59 °C for 30 s, and elongation at 72 °C for 1 min; followed by a final elongation step at 72 °C for 10 min. The PCR products were examined on 1.5% AGE with Genecolor (Gene-Bio) staining.

### 2.3. Identification and Analysis of DEGs

The expression level of each transcript was normalized with the fragments per kilobase of transcript per million mapped reads (FPKM) method [45], which could eliminate the influence of different transcript lengths and sequencing data amounts on the calculation of transcript expression. The *p* value corresponded to differential gene expression at statistically significant levels [50]. DEGs were identified following the standard of *p* ≤ 0.05 and absolute value of log_2_(fold change) ≥ 1. Venn analysis was performed to identify the shared up-regulated and down-regulated genes between AcCK1 vs. AcT1 and AcCK2 vs. AcT2 comparison groups based on the OmicShare platform (www.omicshare.com/tools) (6 April 2021).

### 2.4. GO Categorization and KEGG Pathway Enrichment Analysis of DEGs

GO classification of DEGs was conducted using WEGO software [51]. KEGG pathway annotation was performed with Blastall software against the Kyoto Encyclopedia of Genes and Genomes (KEGG) database (http://www.kegg.jp/) (7 April 2021). Pathways with a corrected *p* value ≤ 0.05 were designated as significantly enriched pathways for DEGs.

### 2.5. Construction and Analysis of Immune-Associated Regulatory Network of DEGs

On the basis of the results of KEGG pathway enrichment analysis, cellular immune pathways (ubiquitin mediated proteolysis, phagosome, autophagy, lysosome, endocytosis, and melanogenesis) as well as humoral immune pathways (MAPK, Jak-STAT, Toll/Imd, and NF-*κ*B signaling pathways) were selected for construction of a regulatory network following the enrichment relationship between DEGs and the above-mentioned immune-associated pathways following our previously described method [52]. The regulatory network was then visualized with Cytoscape v3.2.1 (Cytoscape, Bethasda, MD, USA) [53].

### 2.6. RT-qPCR Validation of DEGs

To verify the reliability of the transcriptome datasets used in this study, nine DEGs (XM_017062027.1, TCONS_00045707, XM_017062123.1, XM_017064466.1, XM_017057571.1, XM_017050556.1, XM_017063843.1, XM_017048437.1, XM_017051020.1) in AcCK1 vs. AcT1 and nine DEGs (XM_017053873.1, XM_017066488.1, TCONS_00045565, XM_017051020.1, TCONS_00009057, XM_017054185.1, XM_017062504.1, XM_017050756.1, XM_017057504.1) in AcCK2 vs. AcT2 were randomly selected for RT-qPCR. The first cDNA strand was synthesized with the SuperScript first-strand synthesis system (Yeasen) according to the protocol. Primers (shown in Table S1) for qPCR were designed utilizing DNAMAN software and synthesized by Sangon Biotech Co., Ltd. (Shanghai). The housekeeping gene *actin* was used as an internal control. The RNA samples used as templates for RNA-seq were the same as those used for RT-qPCR, which was conducted on a QuanStudio Real-Time PCR System (ThemoFisher, Walthem, MA, USA). The 20 µL PCR reaction mixture contained 10 µL SYBR Green dye (Yeasen), 1 µL (10 µmol/L) specific forward primer, 1 µL (10 µmol/L) reverse primer, 1 µL (10 ng/µL) diluted cDNA, and 7 µL RNase free water. Cycling parameters were as follows: 95 °C for 1 min, followed by 40 cycles at 95 °C for 15 s, 55 °C for 30 s, and 72 °C for 45 s. The relative gene expression was calculated using the 2^−^^ΔΔ^^CT^ method [54]. The experiment was carried out times using three independent biological samples.

### 2.7. Statistical Analysis

All statistical analyses were performed using SPSS software (IBM, Armonk, NY, USA) and GraphPad Prism 6.0 software (GraphPad, San Diego, CA, USA). Data were presented as mean ± standard deviation (SD). Statistical analysis was performed using independent-samples *t*-test and one-way ANOVA.

## 3. Results

### 3.1. Effective Infection of A. c. cerana Worker by N. ceranae

**Figure 1:**
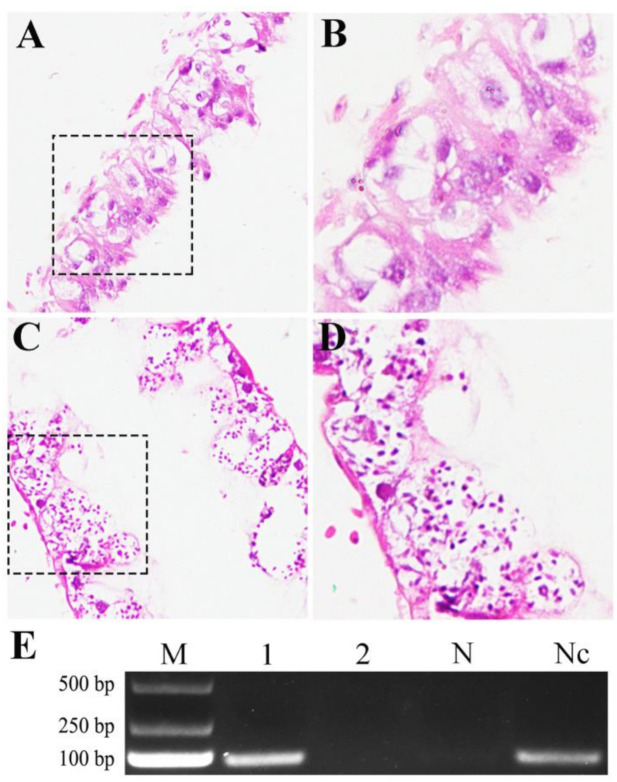
Confirmation of effective infection of *A. c. cerana* worker by *N. ceranae*. (**A**,**B**) Microscopic observation of paraffin section of workers’ midgut at 12 dpi without *N. ceranae* under 200 times (**A**) and 400 times (**B**) amplification. (**C**,**D**) Microscopic observation of paraffin section of workers’ midgut at 12 dpi with *N. ceranae* under 200 times (**C**) and 400 times (**D**) amplification. Black dashed boxes show the selected regions for microscopic observation under 400 times amplification. (**E**) AGE for PCR amplification products from *N. ceranae*-inoculated workers’ midgut and un-inoculated workers’ midgut. Lane M: DNA marker; Lane 1: *A. c. cerana* workers’ midgut at 12 dpi with *N. ceranae*; Lane 2: *A. c. cerana* workers’ midgut at 12 dpi without *N. ceranae*; Lane N: sterile water (negative control); Lane Nc: clean spores of *N. ceranae* (positive control).

Microscopic observation of paraffin sections indicated that no spores were present in epithelial cells of un-inoculated workers’ midgut (Figure 1A,B), while a number of spores were detected in epithelial cells of workers’ midgut at 12 dpi with *N. ceranae* (Figure 1C,D). Additionally, PCR identification demonstrated that the expected fragment (about 76 bp) could be amplified from *N. ceranae*-inoculated midgut, which was in accordance with that amplified from *N. ceranae* spores, whereas no signal fragment could be amplified from un-inoculated midgut (Figure 1E). Together, these results confirmed the effective infection of *A. c. cerana* workers by *N. ceranae*.

### 3.2. DEGs Involved in A. c. cerana Workers’ Midgut Response to N. ceranae Infection

**Figure 2:**
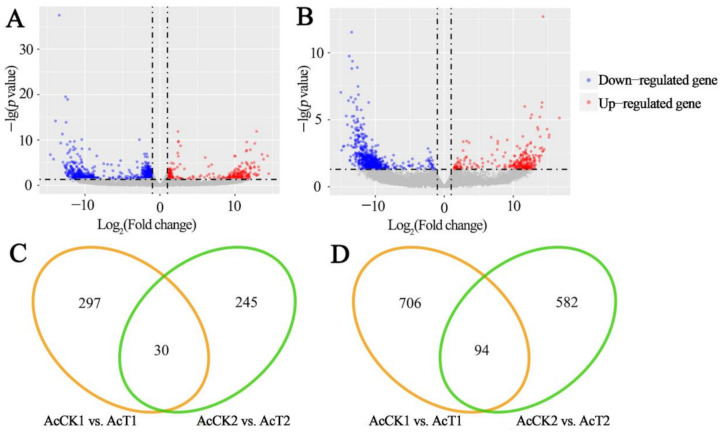
DEGs in *A. c. cerana* workers’ midgut response to *N. ceranae* infection. (**A**,**B**) Valcano plots of DEGs within AcCK1 vs. AcT1 and AcCK2 vs. AcT2 comparison groups. (**C**,**D**) Venn analysis of up-regulated and down-regulated genes within two comparison groups.

In comparison with uninfected workers’ midgut, 1127 DEGs were identified in *N. ceranae*-infected workers’ midgut at 7 dpi, including 327 up-regulated and 800 down-regulated genes (Figure 2A), whereas 951 DEGs were identified in midgut at 10 dpi with *N. ceranae*, including 275 up-regulated and 676 down-regulated genes (Figure 2B). In addition, 30 up-regulated and 94 down-regulated genes were shared by AcCK1 vs. AcT1 and AcCK2 vs. AcT2 comparison groups, whereas the numbers of specific up-regulated (down-regulated) genes were 297 (706) and 245 (582) (Figure 2C,D). The shared up-regulated and down-regulated genes are shown in Table S2, while the specific up-regulated and down-regulated genes are presented in Tables S3 and S4.

**Figure 3:**
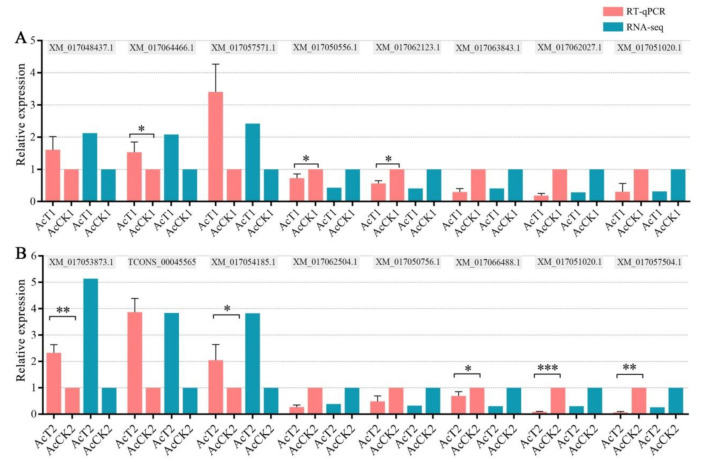
RT-qPCR validation of DEGs in AcCK1 vs. AcT1 (**A**) and AcCK2 vs. AcT2 (**B**) comparison group. * *p* < 0.05; ** *p* < 0.01, *** *p* < 0.001.

Furthermore, RT-qPCR results suggested that the differential expression trends of eight DEGs in AcCK1 vs. AcT1 and eight DEGs in AcCK2 vs. AcT2 were consistent with those in deep sequencing data (Figure 3), which validated the reliability of our transcriptome data.

### 3.3. GO Terms and KEGG Pathways Enriched by Host DEGs

**Figure 4:**
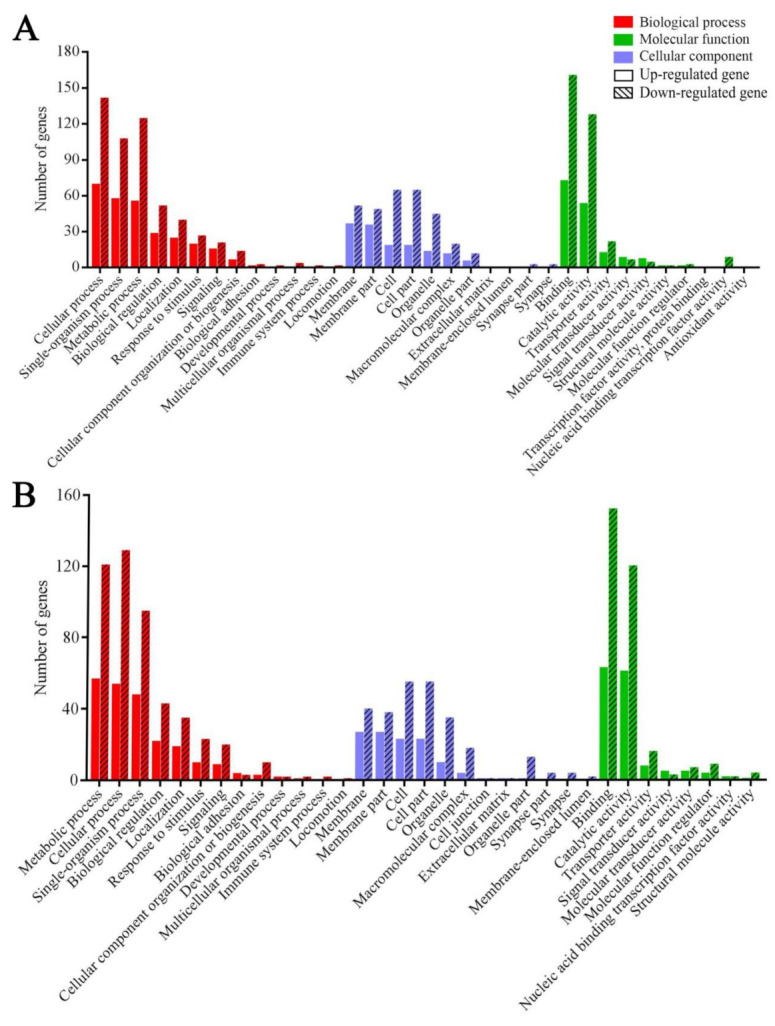
GO classification of DEGs in *A. c. cerana* workers’ midguts at 7 dpi (**A**) and 10 dpi (**B**) with *N. ceranae*.

GO categorization demonstrated that DEGs in the AcCK1 vs. AcT1 comparison group were engaged in 13 biological process-associated functional terms such as cellular processes and single-organism processes, 11 cellular component-associated terms such as membrane parts and cell parts, and 10 molecular function-associated terms such as binding and catalytic activity (Figure 4A); whereas DEGs in the AcCK2 vs. AcT2 comparison group were involved in 33 functional terms relative to biological processes, cellular components, and molecular functions including cellular processes, single-organism processes, biological regulation, binding, and catalytic activity (Figure 4B).

**Figure 5:**
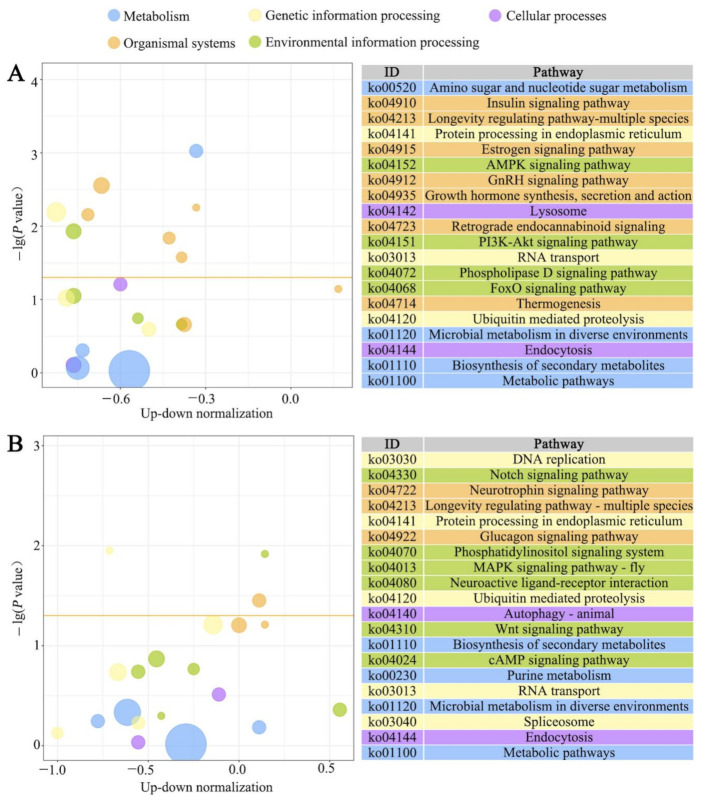
KEGG pathways enriched by DEGs in *A. c. cerana* workers’ midguts at 7 dpi (**A**) and 10 dpi (**B**) with *N. ceranae*.

Moreover, KEGG pathway enrichment analysis showed that DEGs in *N. ceranae*-infected midgut at 7 dpi could be enriched in 231 pathways. Among them, the most abundant ones were biosynthesis of secondary metabolites, protein processing in endoplasmic reticulum, RNA transport, insulin signaling pathway, and AMPK signaling pathway (Figure 5A), whereas DEGs in midgut at 10 dpi with *N. ceranae* could be enriched in 226 pathways, the largest groups of which were biosynthesis of secondary metabolites, protein processing in endoplasmic reticulum, ubiquitin-mediated proteolysis, phosphatidylinositol signaling system, and glucagon signaling pathway (Figure 5B).

### 3.4. Host DEGs Relevant to Cellular and Humoral Immune Pathways

**Figure 6:**
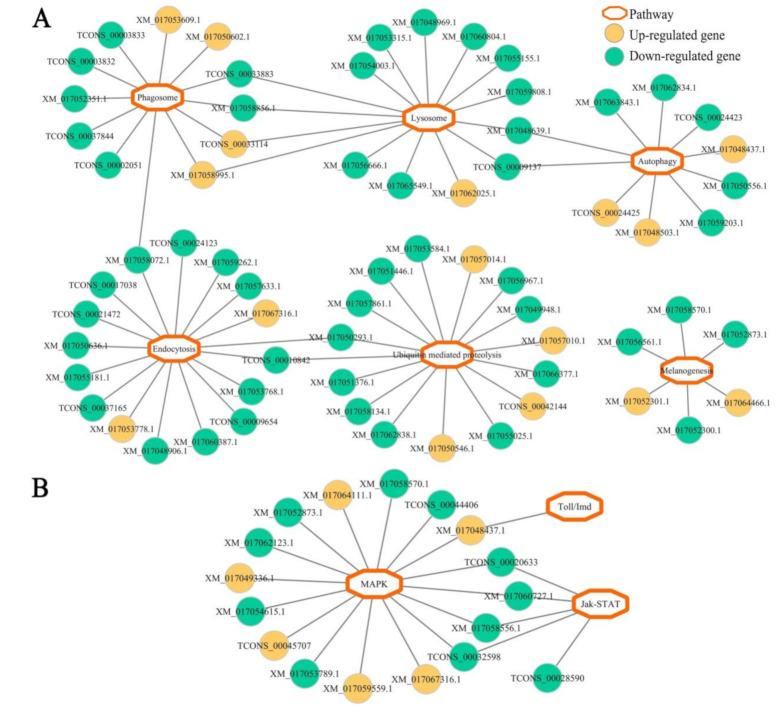
Enrichment network of host DEGs associated with cellular (**A**) and humoral (**B**) immune pathways in AcCK1 vs. AcT1 comparison group.

**Figure 7:**
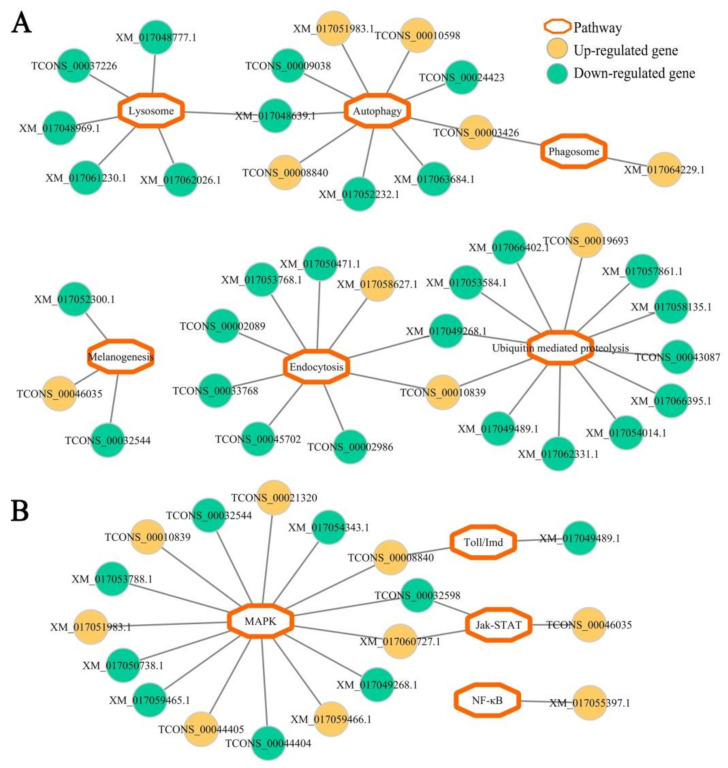
Enrichment network of host DEGs relevant to cellular (**A**) and humoral (**B**) immune pathways in AcCK2 vs. AcT2 comparison group.

Based on the pathway analysis results, DEGs in *N. ceranae*-infected midgut at 7 dpi were found to be involved in six cellular immune pathways (ubiquitin-mediated proteolysis, endocytosis, lysosome, phagosome, autophagy, and melanogenesis) and three humoral immune pathways (MAPK, Jak-STAT, and Toll/Imd signaling pathways) (Figure 6, see also Table S5). Similarly, DEGs in midgut at 10 dpi with *N. ceranae* were also engaged in the aforementioned six cellular immune pathways and four humoral immune pathways (Figure 7, see also Table S6); additionally, one up-regulated gene (XM_017055397.1) was enriched in NF-*κ*B signaling pathway (Figure 7, see also Table S6).

Further investigation of the regulatory network of cellular and humoral immune pathway-associated DEGs revealed that a majority of DEGs in both AcCK1 vs. AcT1 and AcCK2 vs. AcT2 comparison groups were engaged in only one immune pathway (Figure 6 and Figure 7), whereas a small quantity of DEGs were simultaneously involved in two immune pathways. For example, XM_017048639.1 with up-regulated expression in workers’ midgut at 7 dpi was simultaneously enriched in autophagy and lysosome, and TCONS_00032598 was up-regulated in workers’ midgut at 10 dpi and simultaneously enriched in MAPK and Jak-STAT signaling pathways (Figure 6 and Figure 7). Detailed information about cellular and humoral immune pathway-related DEGs in *N. ceranae*-infected *A. c. cerana* workers’ midguts at 7 dpi and 10 dpi is summarized in Tables S7 and S8, respectively.

## 4. Discussion

In our previous work, *A. m. ligustica* workers’ midguts at 7 dpi and 10 dpi with *N. ceranae* were selected for next-generation sequencing based on three major points: (1) the life cycle of *N. ceranae* is approximately 6 d [55]; (2) the number of fungal spores in the infected midgut of *A. m. ligustica* worker continuously increased during a 1–20 d period [56]; (3) the cumulative mortality rate of *N. ceranae*-infected workers was significantly higher than that of uninfected workers at 7 dpi and 10 dpi [52]. We previously performed strand-specific cDNA library-based deep sequencing of un-inoculated midguts and *N. ceranae*-inoculated *A. c. cerana* workers’ midguts at 7 dpi and 10 dpi following the findings from previous studies on *A. mellifera*–*N. ceranae* interaction [29,46,55,56,57]. Here, we first investigated immune response of *A. c. cerana* workers’ midguts to *N. ceranae* infection at a transcriptomic level, and 1127 and 951 DEGs were respectively identified in *N. ceranae*-infected workers’ midguts at 7 dpi and 10 dpi when compared with uninfected workers’ midguts, indicating that overall alteration of host gene expression profile was caused by fungal invasion. Another reason for sequencing midgut samples at the above-mentioned two time points was to better compare *N. ceranae*-response between *A. c. cerana* workers and *A. m. ligustica* workers. Honeybee midgut is a vital site for food digestion, nutrient absorption, detoxification, and immunity, but also the main site for *N. ceranae* proliferation and host-microspodian interaction [58]. As for western honeybee–*N. ceranae* interaction, midgut tissue was usually selected for investigation in previous studies [15,21,58,59]. Therefore, transcriptome data from midguts of *A. c. cerana* workers challenged by *N. ceranae* were believed to more accurately reflect host immune response.

As a eusocial insect, honeybees have evolved both colony-level and individual-level strategies to defense against various pathogens and parasites [60]. Here, we focused on the individual-level immune response of the *A. c. cerana* worker to *N. ceranae* infection. Similarly to vertebrates, insects defend themselves against an array of microbes and parasites by invoking various innate immune responses [61]. In honeybees, cuticle and peritrophic membrane are the physical barriers to combat fungal pathogens. When the first line of defense is pierced, host cellular and humoral immune systems are activated to fight against the invading fungi [62,63,64]. Autophagy literally translates as “eating of self”, which is not only an evolutionarily conserved stress-responsive cytosolic process in both vertebrates and invertebrates, but also an intracellular multistep process responsible for the degradation and recycling of cytoplasmic contents by lysosomal proteases [65,66]. However, little is known about autophagy in honeybee response to infections by various pathogens. Here, three and four up-regulated genes were found to be enriched in autophagy in *A. c. cerana* workers’ midguts at 7 dpi and 10 dpi with *N. ceranae*. Autophagy is relevant to the resistance of mammalian cells to bacteria, viruses, and parasites [67,68,69]. In insects such as *Drosophila melanogaster* and *Aedes aegypti*, autophagy has been proved to be associated with host resistance to bacterial and viral challenges [70,71]. The results indicated that the autophagy pathway may be activated and employed by the host to combat *N. ceranae*. Additionally, in workers’ midgut at 10 dpi, one up-regulated gene encoding phosphatidylinositol 3-kinase catalytic subunit type 3-like isoform X2 [TCONS_00003426, log_2_(fold change) = 12.17] was simultaneously enriched in autophagy and lysosome (Figure 7A), suggestive of the importance of TCONS_00003426 in cellular immune response of the host to *N. ceranae* infection. Moreover, seven and five genes with down-regulation were respectively enriched in autophagy in *N. ceranae*-infected workers’ midguts at 7 dpi and 10 dpi. Although autophagy has been regarded as a cornerstone of host defense and intracellular surveillance, it has been suggested that intracellular bacteria and viruses modify autophagy to enhance their infections [72,73]. This was suggestive of the inhibition of the autophagy pathway in *A. c. cerana* workers’ midgut by *N. ceranae*. Interestingly, in workers’ midgut at 7 dpi, two down-regulated genes encoding lysosomal aspartic protease-like (TCONS_00009137, log_2_(fold change) = −11.16) and lysosomal aspartic protease (XM_017048639.1, log_2_(fold change) = −1.17) were simultaneously enriched in autophagy and lysosome (Figure 6A), implying that TCONS_00009137 and XM_017048639.1, as two key players in host cellular immune response, were likely to be manipulated by *N. ceranae*.

Here, eight down-regulated genes were involved in phagosome in workers’ midgut at 7 dpi, whereas up-regulation of two phagosome-associated genes was observed in workers’ midgut at 10 dpi (Figure 6A and Figure 7A). Phagocytosis is a fundamental innate immune mechanism used by mammals and insects [61]. In honeybees, phagocytosis is one of the most common defense mechanisms to cope with fungal pathogens [62]. The results indicated that the phagosome pathway in host midgut was first inhibited and then markedly activated during *N. ceranae* infection. Comparatively, in the midguts of *A. m. ligustica* workers at 7 dpi and 10 dpi with *N. ceranae*, there was no DEG enriched in phagosome [30]. Together, these findings suggested that the phagosome pathway and corresponding DEGs may play a special role in cellular immune response of *A. c. cerana* workers to *N. ceranae* invasion. Of note, four up-regulated genes were involved in ubiquitin-mediated proteolysis in workers’ midgut at 7 dpi, while more genes (12) enriched in this cellular immune pathway were down-regulated (Figure 6A). A similar situation was detected in the midguts of *A. c. cerana* workers at 10 dpi. As a key mechanism for clearing unneeded or damaged cells, the ubiquitin proteasome degradation system plays a pivotal part in regulating most cellular processes including the cell cycle and immunity [74]. This result demonstrated that ubiquitin-mediated proteolysis in host midgut was suppressed to a large extent by *N. ceranae*. In *A. m. ligustica* workers’ midguts at 7 dpi and 10 dpi with *N. ceranae*, two and two genes involved in ubiquitin-mediated proteolysis were previously found to be up-regulated, respectively, indicative of the activation of this cellular immune pathway during *N. ceranae* infection [30]. As a highly host-dependent fungal parasite, *N. ceranae* is able to induce multidimensional physiological alterations in bee hosts including energetic stress, decreased homing ability, and earlier foraging activity [75,76,77]. Additionally, immune suppression caused by *N. ceranae* infection was observed in western honeybees [15,22,23]. Therefore, these results revealed that cellular immune pathways in *A. c. cerana* workers’ midguts were suppressed by *N. ceranae* to some extent via host–microsporidian interaction.

In this current work, Venn analysis displayed that the numbers of specific up-regulated genes were 297 and 245, which were much less than those (706 and 582) of specific down-regulated genes in AcCK1 vs. AcT1 and AcCK2 vs. AcT2; additionally, only 124 DEGs were shared by the above-mentioned two comparison groups, including 30 up-regulated and 94 down-regulated genes. It is speculated that some DEGs in *A. c. cerana* workers’ midguts continuously exerted key functions during *N. ceranae* invasion, whereas more unique DEGs were involved in host response to fungal infection at various stages. Further, we note that a total of six common genes were enriched in the ubiquitin-mediated proteolysis pathway, and all presented a down-regulated trend in workers’ midguts at both 7 dpi and 10 dpi (Table S2). In addition, an apoptosis-inducing factor 1 encoding gene was obviously down-regulated in workers’ midguts at 7 dpi (log_2_(fold change) = −10.81) and 10 dpi (log_2_(fold change) = −12.19). These results suggested that the aforementioned two cellular immune pathways were suppressed by *N. ceranae* at different stages of fungal infection.

One up-regulated gene was previously discovered to be enriched in apoptosis in the midguts of *A. m. ligustica* workers at 7 dpi and 10 dpi with *N. ceranae*, respectively, suggesting that that apoptosis pathway was activated to combat *N. ceranae* infection [30]. Intriguingly, there was no DEG enriched in apoptosis in this work. Apoptosis plays a primary role in the growth and development of vertebrates and invertebrates [78]. This strategy is also employed by insects to fight against pathogens [79,80]. In western honeybees, *N. ceranae* was shown to suppress apoptosis in host midgut epithelial cells [15,19,28]. However, *N. ceranae*-tolerant *A. mellifera* workers could escape parasitic manipulation of apoptosis [20]. That finding demonstrated that this cellular immune pathway was not engaged in *A. c. cerana* worker midguts responding to *N. ceranae* invasion.

After fungal infection, insect humoral immunity is induced to synthesize AMPs and activate the prophenoloxodase system, which can work together to inactivate or kill the invaders [81]. Currently, most of the knowledge regarding insect humoral immunity is derived from studies associated with on model insects such as *Drosophila* and mosquito. The NF-*κ*B pathway, a conserved signal transduction pathway present in nearly all mammalian cell types, is involved in various biological processes including innate and adaptive immunity, survival, development, proliferation, and cell apoptosis [82,83]. Here, one up-regulated gene was involved in the NF-*κ*B pathway in *N. ceranae*-challenged *A. c. cerana* workers’ midgut at 10 dpi, and one up-regulated gene and one down-regulated gene were enriched in the Toll-like receptor signaling pathway (Figure 7B), whereas in workers’ midgut at 7 dpi, one gene enriched in the Toll-like receptor signaling pathway was found to be up-regulated (Figure 6B). *Drosophila* possess two NF-*κ*B signaling pathways (Toll and Imd) that are uniquely active against different pathogens [84,85,86,87]. The Toll pathway is activated mainly by fungal and Gram-positive bacterial infections and controls in large part the expression of AMPs active against fungal pathogens, whereas the Imd pathway responds mainly to Gram-negative bacteria infections and controls the gene expression of antibacterial peptides [87,88,89]. In the midguts of *A. m. ligustica* workers at both 7 dpi and 10 dpi with *N. ceranae*, one up-regulated gene was enriched in the NF-*κ*B pathway, implying that this humoral immune pathway was activated by *N. ceranae* infection [30]. These results indicated that the NF-*κ*B pathway may play a pivotal role in immune responses of both eastern honeybee workers and western honeybee workers to *N. ceranae* invasion. In addition, five genes enriched in the Jak-STAT pathway were up-regulated in workers’ midgut at 7 dpi, while three DEGs in workers’ midgut at 10 dpi were engaged in this humoral immune pathway, including two up-regulated genes (Figure 6B and Figure 7B). The Jak-STAT pathway was verified to be required for the antiviral response in *Drosophila* [90,91]. Jak-STAT-deficient flies are resistant to fungal and bacterial infections but susceptible to viral infections [92]. This immune pathway was also well-activated in *Anopheles gambiae* responding to bacterial infection [91]. Additionally, similarly to the Toll pathway, the Jak-STAT pathway regulates several other developmental and physiological functions in insects [93]. It has been demonstrated that the Jak-STAT pathway in *A. c. cerana* workers’ midguts was activated due to *N. ceranae* infection. Intriguingly, there was no DEG enriched in the Jak-STAT pathway in *A. m. ligustica* workers’ midguts in response to *N. ceranae* infection [30]. Together, these results demonstrated that different humoral immune responses were employed by *A. c. cerana* workers and *A. m. ligustica* workers to battle the same fungal parasite, *N. ceranae*.

Additional work is needed to decipher the function of key immune genes with differential expression in *A. c. cerana* worker response to *N. ceranae* invasion. That effort can be expected to provide knowledge for the development of effective and environmentally friendly strategies to control bee nosemosis.

## 5. Conclusions

In the present study, cellular and humoral immune responses of *A. c. cerana*e workers’ midguts to *N. ceranae* invasion were comprehensively investigated at a transcriptome-wide level. These results demonstrated that the overall gene expression profile in host midgut was altered by *N. ceranae* invasion and that some host immune pathways such as the autophagy and Jak-STAT pathway were induced to activation during fungal infection, whereas some were suppressed via host–pathogen interaction. Our findings not only offer a basis for clarification of the mechanism underlying the immune response of *A. c. cerana* to *N. ceranae* infection, but also provide novel insights into eastern honeybee–microsporidian interaction.

## Supplementary Materials

The following are available online at https://www.mdpi.com/article/10.3390/insects12080728/s1. Figure S1. Agarose gel electrophoresis for PCR amplification products from the midgut of newly emerged A. c. cerana worker, Table S1. Primers for RT-PCR and RT-qPCR in this research, Table S2. Information about the shared DEGs between AcCK1 vs. AcT1 and AcCK2 vs. AcT2 comparison groups, Table S3. Information about the specific DEGs in AcCK1 vs. AcT1 comparison group, Table S4. Information about the specific DEGs in AcCK2 vs. AcT2 comparison group, Table S5. Summary of cellular and humoral immune pathways enriched by DEGs in AcCK1 vs. AcT1 comparison group, Table S6. Summary of cellular and humoral immune pathways enriched by DEGs in AcCK2 vs. AcT2 comparison group, Table S7. Detailed information about cellular and humoral immune pathway-associated host DEGs at 7 dpi with N. ceranae, Table S8. Detailed information about cellular and humoral immune pathway-associated host DEGs at 10 dpi with N. ceranae.
