# Supplementary material for: Immune Response of Eastern Honeybee Worker to Nosema ceranae Infection Revealed by Transcriptomic Investigation
Source: Insects. 2021 Aug 14;12(8):728. doi: 10.3390/insects12080728 (PMC8396959; doi:10.3390/insects12080728)
Supplement: Supplementary file 1 [file insects-12-00728-s001.zip › Table S1.pdf]

Table S1. Primers for RT-PCR and RT-qPCR in this research.

Gene ID	Primer sequence (5'-3')
XM_017048437.1	F: ACACCAGATTCACCATTC<br>R: ATAAGTTCCATCACCAAGTA
XM_017064466.1	F: GTGAAACTTTTACACCCGCC<br>R: TCCACTATCTCCTCCATCCG
XM_017057571.1	F: ACTTTCAACTCATTTGCCGA<br>R: TGATGTGGTGGTGCTTTCTC
XM_017050556.1	F: TTCGTTTTTACAATAGCCGC<br>R: TTGCCATAACCACCCTTCCC
XM_017062123.1	F: ATGGTATGGTAGTTCCAGAGG<br>R: TGAAGGACGAGAATAAGGTTT
XM_017063843.1	F: GAGTTTTCCCTATTCCAG<br>R: GATGATGTGCTTCCTTGA
XM_017062027.1	F: CTGCTGCGTATGGGGTAA<br>R: GTGGGTTCGGGAAGTTTG
XM_017051020.1	F: AAACATCACCGATTCAACCT<br>R: TTTCCTCCAACAACCACTTC
XM_017053873.1	F: ACCTTTGGCTGAGTTTTTGC<br>R: AACCGTGCTCGTCTTTCTTT
TCONS_00045565	F: GCCGACCTACTTCCCTAT<br>R: CACCCCGTCAGTCTTTCT
XM_017054185.1	F: TGGGTGTTGCTGTCGTTG<br>R: GTGATTTCGGGGCTGGTG
XM_017062504.1	F: CATTCCTAAAGACTCCCA<br>R: GATTTTCCCGAACTGATA
XM_017050756.1	F: ATCAACTAAACCCAGAGAAA<br>R: AAAGCACATAGACGAACACC
XM_017066488.1	F: GATGAAGGCGAAAAGGC<br>R: CCAAAGCACACGGAGAA
XM_017051020.1	F: ACATCACCGATTCAACCT<br>R: TCCTCCAACAACCACTTC
XM_017057504.1	F: GAGAATGCCAAAGAAGGAGA<br>R: GTGATGGAAGAGGGACAAGG
actin	F: GGTTGTTGATAGTGGAGATGG<br>R: CACGACCAGCAATAGGAAT
