# Supplementary material for: Immune Response of Eastern Honeybee Worker to Nosema ceranae Infection Revealed by Transcriptomic Investigation
Source: Insects. 2021 Aug 14;12(8):728. doi: 10.3390/insects12080728 (PMC8396959; doi:10.3390/insects12080728)
Supplement: Supplementary file 1 [file insects-12-00728-s001.zip › Table S5.pdf]

Table S5. Summary of cellular and humoral immune pathways enriched by DEGs in AcCK1 vs AcT1 comparison group.

**Pathway**	**Num of up-regulated gene**	**Num of down-regulated gene**	***p* value**	***q* value**	**Pathway ID**
Ubiquitin mediated proteolysis	4	12	0.255516	0.7690776	ko04120
Phagosome	4	8	0.040119	0.4060341	ko04145
Autophagy	3	7	0.652592	0.9359513	ko04140
Lysosome	3	12	0.061999	0.4889339	ko04142
Endocytosis	2	15	0.781807	0.9616268	ko04144
Melanogenesis	2	4	0.649273	0.9359513	ko04916
MAPK signaling pathway	10	6	0.223438	0.7547242	ko04010
Jak-STAT signaling pathway	0	5	0.11822	0.5796613	ko04630
Toll/Imd signaling pathway	1	0	0.849659	0.9859088	ko04624
