# Supplementary material for: Immune Response of Eastern Honeybee Worker to Nosema ceranae Infection Revealed by Transcriptomic Investigation
Source: Insects. 2021 Aug 14;12(8):728. doi: 10.3390/insects12080728 (PMC8396959; doi:10.3390/insects12080728)
Supplement: Supplementary file 1 [file insects-12-00728-s001.zip › Table S6.pdf]

Table S6. Summary of cellular and humoral immune pathways enriched by DEGs in AcCK2 vs AcT2 comparison group.

**Pathway**	**Num of up-regulated gene**	**Num of down-regulated gene**	***p* value**	***q* value**	**Pathway ID**
Ubiquitin mediated proteolysis	2	10	0.18399	0.986272	ko04120
Phagosome	2	0	0.948736	0.986272	ko04145
Autophagy	4	5	0.307325	0.986272	ko04140
Lysosome	0	6	0.646095	0.986272	ko04142
Endocytosis	2	7	0.924797	0.986272	ko04144
Melanogenesis	1	2	0.825223	0.986272	ko04916
MAPK signaling pathway	7	8	0.293173	0.986272	ko04010
Jak-STAT signaling pathway	2	1	0.257039	0.986272	ko04630
Toll/Imd signaling pathway	1	1	0.351973	0.986272	ko04624
NF-κB signaling pathway	1	0	0.694568	0.986272	ko04064
