# Supplementary material for: Immune Response of Eastern Honeybee Worker to Nosema ceranae Infection Revealed by Transcriptomic Investigation
Source: Insects. 2021 Aug 14;12(8):728. doi: 10.3390/insects12080728 (PMC8396959; doi:10.3390/insects12080728)
Supplement: Supplementary file 1 [file insects-12-00728-s001.zip › Table S7.pdf]

**Table S7.** Detailed information of DEGs involved in immune pathways in AcCK1 vs AcT1 comparison group.

| Gene ID | FPKM in AcCK1 | FPKM in AcCK2 | $Log_2$(Fold change) | *p* value | Nr annotation |
|---|---|---|---|---|---|
| TCONS_00002051 | 33.74 | 13.30666667 | -1.34231 | 0.005845 | Calnexin isoform X3 |
| TCONS_00003832 | 2.39666667 | 0.001 | -11.2268 | 0.048868 | Calreticulin |
| TCONS_00003833 | 113.57 | 41.62666667 | -1.448 | 0.000502 | Calreticulin |
| TCONS_00009137 | 2.28666667 | 0.001 | -11.159 | 0.016938 | Lysosomal aspartic protease-like |
| TCONS_00010842 | 1.37333333 | 0.001 | -10.4235 | 0.013718 | E3 ubiquitin-protein ligase Nedd-4 isoform X3 |
| TCONS_00024423 | 0.62 | 0.001 | -9.27612 | 0.006014 | Inositol 1,4,5-trisphosphate receptor isoform X5 |
| TCONS_00024425 | 0.001 | 0.66 | 9.366322 | 0.022997 | Inositol 1,4,5-trisphosphate receptor isoform X7 |
| TCONS_00033114 | 0.001 | 2.043333333 | 10.99671 | 0.015923 | V-type proton atpase subunit H isoform X3 |
| TCONS_00033883 | 1.91666667 | 0.001 | -10.9044 | 0.014938 | V-type proton atpase 116 kda subunit a isoform 1-like isoform X5 |
| TCONS_00037165 | 0.83666667 | 0.001 | -9.70851 | 0.028903 | Ubiquitin carboxyl-terminal hydrolase 8-like isoform X3 |
| TCONS_00037844 | 1.33666667 | 0.001 | -10.3844 | 0.023167 | V-type proton atpase catalytic subunit A |
| TCONS_00042144 | 0.001 | 3.6 | 11.81378 | 0.004067 | Ubiquitin-conjugating enzyme E2 W-like |
| XM_017048437.1 | 17.8933333 | 38.01666667 | 1.08721 | 0.031943 | Stress-activated protein kinase JNK-like |
| XM_017048503.1 | 0.001 | 1.513333333 | 10.56351 | 0.045198 | Autophagy protein 12-like isoform X2 |
| XM_017048639.1 | 3687.38667 | 1642.233333 | -1.16694 | 0.000298 | LOW QUALITY PROTEIN: lysosomal aspartic protease |
| XM_017048969.1 | 2.84 | 0.001 | -11.4717 | 7.09E-06 | AP-3 complex subunit beta-2 |
| XM_017049948.1 | 25.02 | 10.47 | -1.25682 | 0.003818 | Ubiquitin-protein ligase E3A isoform X1 |
| XM_017050293.1 | 5.2 | 0.001 | -12.3443 | 0.006458 | E3 ubiquitin-protein ligase CBL |
| XM_017050546.1 | 0.001 | 0.866666667 | 9.759333 | 0.040336 | E3 ubiquitin-protein ligase TRIM37-like isoform X5 |
| XM_017050556.1 | 25.5633333 | 10.98 | -1.2192 | 0.018156 | Ribosomal protein S6 kinase beta-1-like |
| XM_017050602.1 | 1310.72667 | 2990.593333 | 1.190065 | 0.000229 | Tubulin beta-1 chain |
| XM_017051376.1 | 18.32 | 8.94 | -1.03507 | 0.017749 | DNA damage-binding protein 1 |
| XM_017051446.1 | 35.0366667 | 11.01333333 | -1.66961 | 0.027002 | E3 ubiquitin-protein ligase synoviolin A isoform X2 |
| XM_017052301.1 | 0.01666667 | 1.026666667 | 5.944858 | 0.018509 | Adenylate cyclase type 3 isoform X1 |
| XM_017052351.1 | 9.66 | 0.001 | -13.2378 | 4.92E-10 | Integrin beta-nu-like isoform X1 |
| XM_017052873.1 | 1.22333333 | 0.001 | -10.2566 | 0.04281 | Protein kinase C isoform X2 |
| XM_017053315.1 | 105.6 | 49.81666667 | -1.08391 | 0.001722 | Prosaposin |

| | | | | | |
|---|---|---|---|---|---|
| XM_017053584.1 | 13.7133333 | 3.38 | -2.02048 | 0.000785 | Ubiquitin conjugation factor E4 B isoform X1 |
| XM_017053609.1 | 0.001 | 3.54 | 11.78953 | 0.03796 | Syntaxin-18 |
| XM_017053778.1 | 0.001 | 1.776666667 | 10.79496 | 0.033052 | Actin-related protein 2/3 complex subunit 4 |
| XM_017054003.1 | 8.42333333 | 2.746666667 | -1.61671 | 0.047938 | Lysosomal alpha-mannosidase isoform X1 |
| XM_017055025.1 | 12.7266667 | 6.143333333 | -1.05076 | 0.02031 | E3 ubiquitin-protein ligase HUWE1 isoform X2 |
| XM_017055155.1 | 23.31 | 11.39 | -1.03318 | 0.038591 | AP-1 complex subunit beta-1 isoform X2 |
| XM_017056666.1 | 39.04 | 12.81 | -1.60768 | 9.83E-05 | Cysteine proteinase |
| XM_017056967.1 | 12.71 | 5.39 | -1.23761 | 0.042383 | E3 ubiquitin-protein ligase RFWD2-like isoform X1 |
| XM_017057010.1 | 0.001 | 1.93 | 10.91439 | 0.017463 | Ubiquitin conjugation factor E4 A |
| XM_017057014.1 | 0.001 | 1.846666667 | 10.85071 | 0.030686 | Rho-related BTB domain-containing protein 1 isoform X1 |
| XM_017057861.1 | 1.95666667 | 0.001 | -10.9342 | 0.001713 | (E3-independent) E2 ubiquitin-conjugating enzyme UBE2O |
| XM_017058072.1 | 11.27 | 4.67 | -1.27099 | 0.043461 | Hepatocyte growth factor-regulated tyrosine kinase substrate |
| XM_017058134.1 | 42.56 | 20.78333333 | -1.03407 | 0.008534 | Cullin-2-like isoform X1 |
| XM_017058856.1 | 3.58 | 0.001 | -11.8057 | 0.000114 | V-type proton atpase subunit H isoform X3 |
| XM_017058995.1 | 155.673333 | 331.4033333 | 1.090066 | 0.012602 | PV-type proton atpase 116 kda subunit a-like isoform X2 |
| XM_017059203.1 | 1.74333333 | 0.001 | -10.7676 | 0.000383 | Hamartin-like isoform X2 |
| XM_017059808.1 | 16.2 | 6.01 | -1.43056 | 0.02703 | Beta-galactosidase-like |
| XM_017060804.1 | 25.59 | 10.29 | -1.31434 | 0.010672 | Cystinosin homolog isoform X1 |
| XM_017062025.1 | 0.00666667 | 2.066666667 | 8.276124 | 0.045591 | Major facilitator superfamily domain-containing protein 8 isoform X1 |
| XM_017062834.1 | 4.82 | 0.001 | -12.2348 | 0.004217 | Ubiquitin-like modifier-activating enzyme atg7 isoform X1 |
| XM_017062838.1 | 0.92666667 | 0.001 | -9.85591 | 0.034219 | Kelch-like ECH-associated protein 1 |
| XM_017063843.1 | 50.84 | 20.65666667 | -1.29936 | 0.002389 | 5'-AMP-activated protein kinase catalytic subunit alpha-2 |
| XM_017064466.1 | 197.023333 | 410.3666667 | 1.058547 | 0.001751 | Armadillo segment polarity protein isoform X1 |
| XM_017065549.1 | 25.45 | 12.51666667 | -1.02382 | 0.047317 | Heparan-alpha-glucosaminide N-acetyltransferase-like isoform X2 |
| XM_017066377.1 | 8.82 | 2.456666667 | -1.84408 | 0.003262 | Dentin sialophosphoprotein-like |
| XM_017067316.1 | 0.13333333 | 2.816666667 | 4.400879 | 0.040002 | Arrestin red cell isoform X2 |
| TCONS_00020633 | 2.756667 | 0.001 | -11.4287 | 0.012565 | Rotein E(sev)2B [Acromyrmex echinatior] |
| TCONS_00028590 | 1.64 | 0.001 | -10.6795 | 0.000971 | Tyrosine-protein kinase hopscotch-like isoform X5 |

| | | | | | |
|---|---|---|---|---|---|
| TCONS_00032598 | 1.423333 | 0.001 | -10.4751 | 0.00199 | Protein son of sevenless isoform X1 |
| TCONS_00044406 | 0.506667 | 0.001 | -8.98489 | 0.001626 | Proto-oncogene tyrosine-protein kinase ROS-like isoform X3 |
| TCONS_00045707 | 30.64333 | 77.62667 | 1.340979 | 0.007133 | Insulin-like receptor-like isoform X1 |
| XM_017049336.1 | 0.001 | 0.913333 | 9.834998 | 0.02168 | ETS-like protein pointed isoform X1 |
| XM_017053789.1 | 20.42333 | 7.586667 | -1.42868 | 0.00364 | Protein eiger isoform X2 |
| XM_017054615.1 | 16.20667 | 6.813333 | -1.25015 | 0.039636 | Ribosomal protein S6 kinase 2 beta isoform X1 |
| XM_017058556.1 | 1.243333 | 0.001 | -10.28 | 0.021786 | Protein son of sevenless |
| XM_017058570.1 | 306.57 | 153.05 | -1.00221 | 0.003022 | Calmodulin [Daphnia magna] |
| XM_017059559.1 | 0.001 | 1.4 | 10.45121 | 0.0415 | Ras gtpase-activating protein 3 isoform X1 |
| XM_017060727.1 | 1.82 | 0.001 | -10.8297 | 0.006208 | Tyrosine-protein phosphatase corkscrew-like isoform X1 |
| XM_017064111.1 | 0.001 | 0.263333 | 8.040746 | 0.042733 | Proto-oncogene tyrosine-protein kinase ROS isoform X3 |
| TCONS_00009654 | 14.74667 | 6.973333 | -1.08047 | 0.032123 | Endophilin-A isoform X14 |
| TCONS_00017038 | 17.59667 | 8.65 | -1.02453 | 0.015064 | Golgi-specific brefeldin A-resistance guanine nucleotide exchange factor 1-like |
| TCONS_00021472 | 2.673333 | 0.001 | -11.3844 | 0.01466 | Actin-related protein 2/3 complex subunit 4 |
| TCONS_00024123 | 3.886667 | 0.316667 | -3.6175 | 0.039663 | F-actin-capping protein subunit alpha-like |
| XM_017048906.1 | 13.51 | 3.9 | -1.79248 | 0.001651 | Atypical protein kinase C-like |
| XM_017050636.1 | 10.85 | 4.21 | -1.3658 | 0.032246 | Partitioning defective 3 homolog |
| XM_017052300.1 | 0.846667 | 0.001 | -9.72565 | 0.00036 | Adenylate cyclase type 3 isoform X2 |
| XM_017053768.1 | 1 | 0.001 | -9.96578 | 0.007915 | Zinc finger FYVE domain-containing protein 16 isoform X2 |
| XM_017055181.1 | 7.866667 | 2.606667 | -1.59355 | 0.034012 | Phospholipase D2 isoform X1 |
| XM_017056561.1 | 1.003333 | 0.001 | -9.97059 | 0.031852 | Adenylate cyclase type 9 isoform X1 |
| XM_017057633.1 | 7.313333 | 3.046667 | -1.2633 | 0.038971 | WASH complex subunit FAM21 |
| XM_017058570.1 | 306.57 | 153.05 | -1.00221 | 0.003022 | Calmodulin |
| XM_017059262.1 | 11.49667 | 2.473333 | -2.21669 | 0.001349 | Epidermal growth factor receptor substrate 15-like 1 isoform X2 |
| XM_017060387.1 | 35.40333 | 13.25333 | -1.41753 | 0.003262 | Stromal membrane-associated protein 1 |
