# Supplementary material for: Immune Response of Eastern Honeybee Worker to Nosema ceranae Infection Revealed by Transcriptomic Investigation
Source: Insects. 2021 Aug 14;12(8):728. doi: 10.3390/insects12080728 (PMC8396959; doi:10.3390/insects12080728)
Supplement: Supplementary file 1 [file insects-12-00728-s001.zip › Table S8.pdf]

Table S8. Summary of DEGs involved in immune pathways of AcCK2 vs AcT2 comparison group.

Gene ID	FPKM in AcCK1	FPKM in AcCK2	Log_2(Fold change)	*p* value	Nr annotation
TCONS_00002089	2.543333	0.001	-11.3125	0.008495	Rabenosyn-5-like
TCONS_00002986	2.923333	0.001	-11.5134	0.022239	Stromal membrane-associated protein 1-like
TCONS_00003426	0.001	4.593333	12.16533	0.006197	Phosphatidylinositol 3-kinase catalytic subunit type 3-like isoform X2
TCONS_00008840	0.001	14.33	13.80675	0.007599	Stress-activated protein kinase JNK isoform X6
TCONS_00009038	3.45	0.001	-11.7524	0.045521	Autophagy protein 12-like isoform X2
TCONS_00010598	0.001	7.063333	12.78613	0.013152	Ras-related GTP-binding protein D-like isoform X1
TCONS_00010839	0.001	4.486667	12.13143	0.009901	E3 ubiquitin-protein ligase Nedd-4-like isoform X6
TCONS_00019693	0.001	4.053333	11.98489	0.048835	E3 ubiquitin-protein ligase parkin-like isoform 1
TCONS_00024423	0.926667	0.001	-9.85591	0.002506	Inositol 1,4,5-trisphosphate receptor isoform X5
TCONS_00032544	0.986667	0.001	-9.94642	0.046584	Csa-calmodulin 6, partial
TCONS_00033768	3.093333	0.001	-11.5949	0.031246	Epidermal growth factor receptor substrate 15-like 1-like isoform X2
TCONS_00037226	1.633333	0.001	-10.6736	0.016125	Cystinosin homolog isoform X1
TCONS_00043087	2.686667	0.001	-11.3916	0.00793	Anaphase-promoting complex subunit 7 isoform X2
TCONS_00045702	2.973333	0.001	-11.5379	0.006572	Maspardin-like isoform X3
TCONS_00046035	0.13	14.47	6.798409	0.000204	LOW QUALITY PROTEIN: histone acetyltransferase p300
XM_017048639.1	2369.72	577.61	-2.03655	0.002655	LOW QUALITY PROTEIN: lysosomal aspartic protease
XM_017048777.1	1.656667	0.001	-10.6941	0.0303	Lipase 3-like isoform X1
XM_017048969.1	1.186667	0.001	-10.2127	0.016024	AP-3 complex subunit beta-2
XM_017049268.1	4.163333	0.001	-12.0235	0.005045	E3 ubiquitin-protein ligase Nedd-4 isoform X1
XM_017049489.1	1.33	0.001	-10.3772	0.040284	Ubiquitin-conjugating enzyme E2 N
XM_017050471.1	13.11667	0.001	-13.6791	0.010258	Actin-related protein 2/3 complex subunit 5
XM_017051983.1	236.58	680.7433	1.524783	0.022809	3-phosphoinositide-dependent protein kinase 1
XM_017052232.1	2.246667	0.001	-11.1336	0.006201	Autophagy-related protein 13 isoform X1
XM_017052300.1	0.66	0.001	-9.36632	0.008586	Adenylate cyclase type 3 isoform X2
XM_017053584.1	14.00667	0.001	-13.7738	1.76E-10	Ubiquitin conjugation factor E4 B isoform X1

XM_017053768.1	0.696667	0.001	-9.44432	0.015373	Zinc finger FYVE domain-containing protein 16 isoform X2
XM_017054014.1	3.54	0.001	-11.7895	0.001764	Ubiquitin-protein ligase E3C isoform X2
XM_017057861.1	4.076667	0.001	-11.9932	0.000115	(E3-independent) E2 ubiquitin-conjugating enzyme UBE2O
XM_017058135.1	1.94	0.001	-10.9218	0.006904	Cullin-2-like isoform X2
XM_017058627.1	0.001	3.06	11.57932	0.03765	EH domain-containing protein 3
XM_017061230.1	16.81333	0.001	-14.0373	2.34E-05	Lipopolysaccharide-induced tumor necrosis factor-alpha factor-like
XM_017062026.1	4.926667	0.001	-12.2664	0.003857	Major facilitator superfamily domain-containing protein 8 isoform X3
XM_017062331.1	2.41	0.001	-11.2348	0.006846	Anaphase-promoting complex subunit 4 isoform X2
XM_017063684.1	1.913333	0.001	-10.9019	0.027655	Cysteine protease ATG4B-like isoform X3
XM_017064229.1	0.001	2.513333	11.29539	0.039606	Tubulin alpha chain-like
XM_017066395.1	2.066667	0.001	-11.0131	0.00704	Dentin sialophosphoprotein-like
XM_017066402.1	1.016667	0.001	-9.98963	0.006007	Dentin sialophosphoprotein-like
TCONS_00021320	0.001	14.28667	13.80238	0.012103	Calcineurin subunit B type 2-like
TCONS_00032598	0.993333	0.001	-9.95613	0.024459	Protein son of sevenless isoform X1
TCONS_00044404	0.47	0.001	-8.87652	0.012638	Proto-oncogene tyrosine-protein kinase ROS isoform X2
TCONS_00044405	0.001	0.42	8.714246	0.049728	Proto-oncogene tyrosine-protein kinase ROS-like isoform X2
XM_017050738.1	1.33	0.001	-10.3772	0.010014	Exportin-1
XM_017053788.1	2.226667	0.001	-11.1207	0.004839	Protein eiger isoform X1
XM_017054343.1	3.713333	0.001	-11.8585	5.12E-05	Mitogen-activated protein kinase kinase kinase 15 isoform X1
XM_017059465.1	10.72333	0.001	-13.3885	3.31E-05	Serine/threonine-protein kinase 3 isoform X2
XM_017059466.1	0.001	4.196667	12.03503	0.021818	Serine/threonine-protein kinase 3 isoform X3
XM_017060727.1	0.001	3.03	11.5651	0.029979	Tyrosine-protein phosphatase corkscrew-like isoform X1
