# Supplementary figures and images for: Immune Response of Eastern Honeybee Worker to Nosema ceranae Infection Revealed by Transcriptomic Investigation
Source: Insects. 2021 Aug 14;12(8):728. doi: 10.3390/insects12080728 (PMC8396959; doi:10.3390/insects12080728)

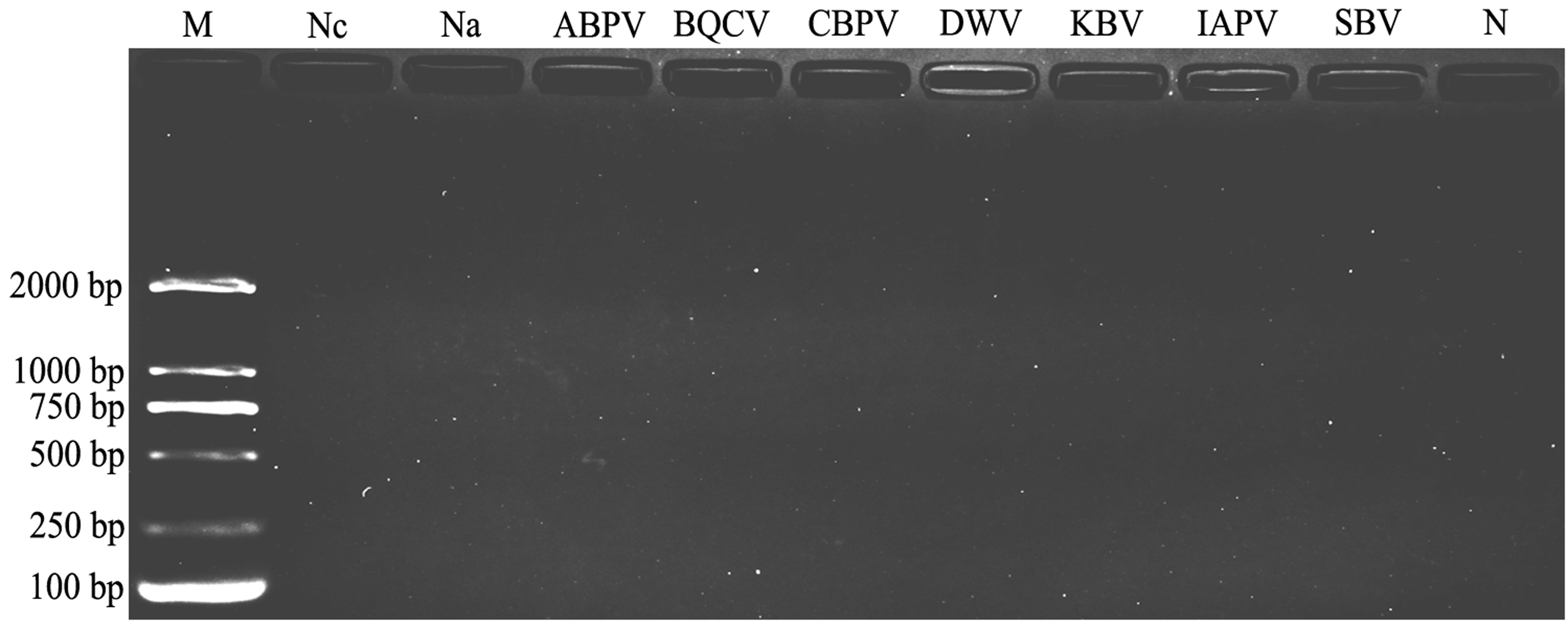

Supplement: Supplementary file 1 [file insects-12-00728-s001.zip › Figure S1.tif]
